# Evaluation of *Artemisia dubia* folium extract-mediated immune efficacy through developing a murine model for acute and chronic stages of atopic dermatitis

**DOI:** 10.1186/s42826-024-00201-x

**Published:** 2024-04-07

**Authors:** Manju Acharya, Ravi Gautam, SuJeong Yang, JiHun Jo, Anju Maharjan, DaEun Lee, Narayan Prasad Ghimire, ByeongSun Min, ChangYul Kim, HyoungAh Kim, Yong Heo

**Affiliations:** 1 Department of Health and Safety, Graduate School, Daegu Catholic University, Gyeongsan-Si, Gyeongbuk Province 38430 Republic of Korea; 2 Central Department of Botany, Tribhuvan University, Kathmandu, Nepal; 3 College of Pharmacy, Daegu Catholic University, Gyeongsan-Si, Gyeongbuk Province 38430 Republic of Korea; 4 Department of Toxicology, Graduate School, Daegu Catholic University, Gyeongsan-Si, Gyeongbuk Province 38430 Republic of Korea; 5 Department of Preventive Medicine, College of Medicine, The Catholic University of Korea, Seoul, 06591 Republic of Korea

**Keywords:** Atopic dermatitis, Murine model, *Artemisia dubia*, Cytokines, Immunoglobulins

## Abstract

**Background:**

Atopic dermatitis (AD) is a biphasic type of skin inflammation characterized by a predominance of type-2 (T_H_2) and type-1 (T_H_1) helper T cell-biased immune responses at the acute and persistent chronic phases, respectively. The present study was aimed to evaluate the efficacy of *Artemisia dubia* folium extract (ADFE) on AD-like skin lesions through developing a murine model for acute and chronic stages of AD. To induce acute phase AD, the dorsal skin of BALB/c mice was sensitized twice a week with 1% 2, 4-dinitrochlorobenzene (DNCB), followed by challenge (twice) in the following week with 0.2% DNCB. To induce persistent chronic AD, some mice were challenged twice a week for 4 more weeks. After the second challenge, the dorsal skin was exposed to 3% ADFE (five times per week) for 2 weeks (acute phase) or 4 weeks (persistent chronic phase).

**Results:**

The paradigm of T_H_2 or T_H_1 predominance at the acute and chronic phase, respectively, was observed in this mouse model. During the acute phase, we observed an increased IL-4/IFN-γ ratio in splenic culture supernatants, an increased IgG1/IgG2a ratio in serum, and elevated serum IgE levels; however, the skew toward T_H_2 responses was diminished during the chronic stage. Compared with vehicle controls, ADFE reduced the IL-4/IFN-γ and IgG1/IgG2a ratios in acute AD, but both ratios increased during the chronic stage.

**Conclusions:**

Our results suggest that ADFE concomitantly suppresses the T_H_2 predominant response in acute AD, as well as the T_H_1 predominant response in chronic AD. Thus, ADFE is a candidate therapeutic for AD.

**Supplementary Information:**

The online version contains supplementary material available at 10.1186/s42826-024-00201-x.

## Background

Atopic dermatitis (AD) is caused by progressive inflammation of the skin, giving rise to pruritic eczematous skin lesions [1]. The clinical manifestations of AD arise through interactions between genetic, environmental, and immunological factors [2]. The primary defect in skin barrier function allows penetration by external antigens, which interact with local antigen-presenting cells and immune effector cells, thereby triggering a systemic immune response [3]. The immunological characteristics of AD differ according to whether the immune response is driven primarily by type-1 helper T lymphocytes (T_H_1) or type-2 helper T lymphocytes (T_H_2) [4]. Early phase acute AD (aAD) is characterized by erythema, eczema, and pruritus, all caused by a predominant T_H_2-mediated immune response via increased production of interleukin (IL)-4, IL-5, and IL-13, and a decrease in production of IL-12 and interferon-gamma (IFN-γ) [5]. IL-4 secreted by T_H_2 cells, mast cells, or other constituent cells triggers B cell isotype switching to secrete IgE and IgG1 [6]. By contrast, persistent chronic AD (cAD) is characterized by dry, thickened, and lichenified skin, and is driven primarily by a T_H_1-mediated immune response associated with production of IFN-γ and IL-12 [4, 7].

Currently, few therapeutic or preventive measures have a positive effect on AD. Maintenance of epidermal homeostasis and inhibition of inflammatory responses using phototherapy, monoclonal antibodies, steroids, or calcineurin inhibitors are common practices worldwide [8, 9], but these approaches have several side effects [10]. Therefore, alternative medicinal trials using natural ingredients such as herbal extracts have been undertaken to identify substances that prevent or treat AD with minimal side effects [11].

Herbal products have been used worldwide for a very long time to cure and prevent skin inflammation [12–14]. Traditionally, *Artemisia dubia* has been used to treat microorganism infection including parasitic disease, cancerous diseases, or skin wounds in South Asia countries [15–18]. Different parts of the Artemisia dubia plant contain triterpenoids (calotropoleanyl ester, α -amyrin), various very-long-chain fatty acids (e.g. nonacosanoic acid, docosanoic acid, tetracosanoic acid), glycerols (e.g. 1-(O-tricosanoyl) glycerol, 1-(O-pentacosanoyl) glycerol, β-sistosterol, and flavonoids [19, 20]. Flavonoids of medicinal plant origin exert anti-inflammatory effects both in vitro and in vivo [21]. Although different species of *Artemisia* (*A. apiacea or A. argyi*) ameliorate AD-like skin lesions in mice [22, 23], no report to date has examined the immunomodulatory potency of *Artemisia dubia* in the context of the balance between T_H_2 or T_H_1 responses during the acute or chronic phases of AD.

The present study aimed to evaluate the ability of *Artemisia dubia* folium extract (ADFE) to alleviate AD-like skin lesions through developing a murine model for acute and chronic stages of AD, which was generated by dermal application of 2,4-dinitrochlorobenzene (DNCB). Then, both cell-mediated and humoral immune responses were evaluated in the context of T_H_2 or T_H_1 predominance during the acute and chronic phases. The histopathological and immunologic features of the DNCB murine model are similar to those in humans with AD, showing predominance of T_H_2-mediated humoral and cellular immune response during the acute phase after challenge with DNCB for 2 weeks [24–26]. In addition, we examined the efficacy of ADFE during the chronic phase, which was induced experimentally by additional DNCB challenge for a further 4 weeks (i.e., 6 weeks in total).

## Methods

### Animals and experimental protocols

Male BALB/c mice (4 weeks of age; 15–18 g) were purchased from a local animal supplier of laboratory animals (Daehan BioLink, Korea). The mice were housed in a controlled environment (temperature 22 ± 2 °C; relative humidity 50 ± 5%; 12 h light–dark cycle) in a laminar flow cabinet and had access to standard rodent chow and autoclaved distilled water ad libitum. All animal maintenance and experimental procedures were conducted in accordance with the National Research Council’s Guide for the Care and Use of Laboratory Animals [27] and were approved by Daegu Catholic University's Institutional Animal Care and Use Committee (IACUC-2018–050). After acclimatization for 1 wk, the mice were randomly allocated into eight groups of five mice per group: Group 1 comprised aAD mice treated with ADFE (aAD( +)/ADFE); Group 2 comprised non-aAD mice treated with ADFE (aAD(-)/ADFE); Group 3 comprised aAD mice treated with 10% ethanol (EtOH), the vehicle used to dissolve ADFE (aAD( +)/EtOH); Group 4 comprised non-aAD mice treated with 10% EtOH (aAD(-)/EtOH); Group 5 comprised aAD not treated at all after aAD induction (aAD( +)); Group 6 comprised non-aAD mice not treated at all (aAD(-)); Group 7 comprised cAD mice treated with ADFE (cAD( +)/ ADFE); and Group 8 comprised cAD mice treated with 10% EtOH (cAD( +)/ EtOH).

### Preparation of *Artemisia dubia* folium extract

*Artemisia dubia* folium was collected in Rimuwa region (27.9973°N, 83.4483°E) of Nepal, and confirmed by the Central Department of Botany, Tribhuvan University, Nepal and verified by the Nepalese Government, Department of Plant Resources (DPR, NPRL-1, 074/76 522) prior to arrival in the Republic of Korea. The dried leaves were extracted with tenfold (w/w) 80% ethanol at a temperature of 55 ± 2 °C using a reflux extractor (MS-DMS634, Thermoline Scientific, Australia). The extracted solution was filtered through Whatman number 2 filter paper (Sigma-Aldrich, St Louis, MO, USA) with a pore size of 8 μm, followed by concentration in a rotary evaporator (SB-1200, Eyela, China) at 55 ± 2 °C. The concentrated extract was then lyophilized (SFDSM12, Samwon, Korea). The ADFE was dissolved in 10% ethanol vehicle to a final concentration of 3% immediately before application to mice, and this single concentration has been used throughout all ADFE treatments. The amount of endotoxin in the ADFE was examined using a Kinetic Chromogenic Limulus Amebocyte Lysate assay (Lonza, Switzerland). The endotoxin concentration was below the level of detection.

### Induction of AD-like skin lesions in mice, and administration of the extract

**Fig. 1:**
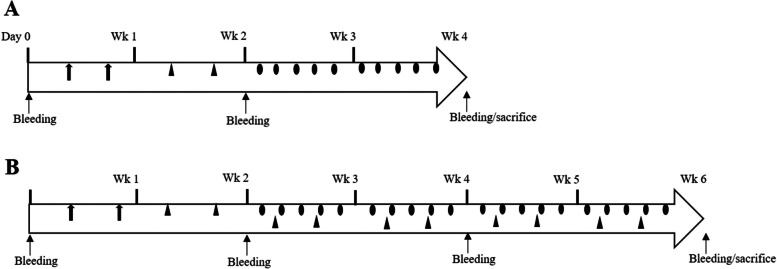
Schematic diagram showing induction of acute atopic dermatitis (**A**) and chronic atopic dermatitis (**B**) in mice. Sensitization with 1% 2,4-dinitrochlorobenzene (DNCB) is indicated by ⬆, challenge with 0.2% DNCB by ▴, and application of 3% *Artemisia dubia* folium extract or vehicle (10% ethanol) by ●

AD was induced as described previously [13, 25, 28]. Briefly, on the day before sensitization, the dorsal skin surface was shaved from the shoulder to the hip (2 × 4 cm) using an electric clipper. Mice were sensitized by two applications (100 μl each) of 1% DNCB or vehicle (acetone: olive oil 4:1; AOO) within 1 week. The mice were then challenged twice with 100 μl of 0.2% DNCB or vehicle on the following week. To induce chronic AD, mice were challenged repeatedly for 5 weeks. ADFE (3%, 200 μl/treatment) or vehicle was applied five times per week for 2 weeks to mice in the early aAD group and for 4 weeks in the cAD group, starting 2 days after the second DNCB challenge (Fig. 1).

### Haematology and histopathology

Cardiac blood was collected from mice at the time of sacrifice (Day 28 for the aAD groups and Day 42 for the cAD groups) through isoflurane (Hana Pharm Co., Korea) inhalation, and hematologic analyses were performed immediately. The percentages of total white blood cells (WBC), neutrophils, lymphocytes, monocytes, eosinophils, and basophils were calculated by an automated hematology analyzer (ADIVA 2120, Siemens, Germany). Treated skin Sects. (2 × 4 cm) were sampled aseptically and fixed in 10% neutral buffered formalin solution. The skin segments were stained with hematoxylin and eosin prior to histopathological analysis, to observe epidermal hyperplasia and inflammatory cell infiltration. Histopathological observation was undertaken by a certified pathologist at the Preclinical Research Center, Daegu Gyeongbuk Medical Innovation Foundation, Republic of Korea. Epidermal thickness was measured by a slide scanner following conversion into digital images.

### Measurement of splenic T cell cytokines

After sacrifice, the spleen was collected aseptically, and a single cell suspension was prepared. Splenic T cells were stimulated in vitro with immobilized anti-CD3 for 48 h in a 37 °C/5% CO_2_ incubator. Culture supernatants were harvested following stimulation and used for evaluating the levels of various cytokines (IFN-γ, IL-4, IL-17, and tumor necrosis factor (TNF)-α) using sandwich ELISA kits (BD Biosciences, Franklin Lakes, New jersey, USA).

### Measurement of serological parameters

Blood was collected from the periorbital plexus under isoflurane anesthesia 1 day before the first sensitization, 1 day after the second challenge, and 1 day after the sixth challenge, and by cardiac puncture at the time of sacrifice. Serum was prepared and used for evaluating levels of IgE, IgG1, and IgG2a by sandwich ELISA, as described elsewhere [28]. Serum histamine levels were measured using a histamine ELISA kit (LDN, Nordhorn, Germany).

### Statistical analyses

All statistical analyses were performed using Sigma Plot 14.0 (Systat Software Inc, San Jose, CA, USA). Depending on the normality of the data, the statistical significance of differences among subgroups with aAD and cAD was tested by one-way analysis of variance (ANOVA) or the Kruskal–Wallis test, Holm-sidak pairwise comparison, or Tukey’s test. Student’s t-test or Mann–Whitney rank-sum tests were performed to determine the statistical differences between the two groups. A *p*-value < 0.05 was considered significant.

## Results

### Effect of ADFE on development of AD-like skin lesions

Application of DNCB to the dorsal skin of mice for 2 weeks induced characteristics typical of early aAD, including skin dryness followed by erythema, eczema, exudation, and pruritus (Additional Fig. 1A). Application of 3% ADFE to the dorsal skin for 2 weeks after induction of AD (aAD( +)/ADFE) led to an improvement in the condition of the skin (Additional Fig. 1B). Similarly, application of DNCB for 6 weeks led to dry, thickened, and lichenified skin with fibrotic papules, characteristics typical of cAD (Additional Fig. 1C). However, cAD( +)/ADFE mice showed no prominent improvement in skin lesions after 4 weeks application of ADFE (Additional Fig. 1D).

### Histopathological or haematological changes after treatment with ADFE

**Fig. 2:**
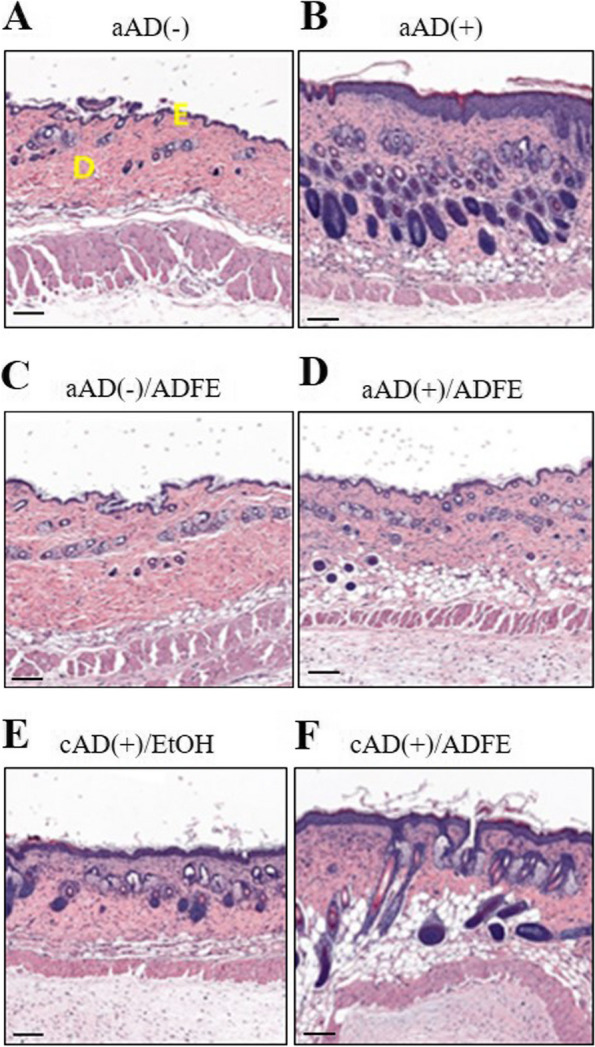
Representative histopathological images of skin from mice treated ADFE or vehicle. Two mouse skin samples per group were stained with hematoxylin and eosin (scale bar = 50 μm, 100X magnification). The aAD(-) and aAD(-)/ADFE showed no evidence of acute atopic dermatitis induction, regardless of ADFE treatment. The aAD( +) and aAD( +)/ADFE groups showed induction of acute atopic dermatitis regardless of ADFE treatment. The cAD( +)/EtOH and cAD( +)/ADFE groups were exposed to 10% ethanol or ADFE. The yellow letters E and D in left panel of (A) denote the epidermal and dermal layer, respectively. ADFE, *Artemisia dubia* folium extract; aAD(-), no acute atopic dermatitis induction; aAD( +), aAD induction; aAD(-)/ADFE, no aAD induction but ADFE treatment; aAD( +)/ADFE, aAD induction with ADFE treatment; cAD( +)/EtOH, chronic AD induction with 10% EtOH application; cAD( +)/ADFE, cAD induction with ADFE treatment

DNCB caused epidermal hyperplasia and inflammatory cell infiltration typical of AD skin (Fig. 2B). Following application of ADFE to the aAD( +)/ADFE group, epidermal thickness (26.1 ± 7.0 μm; Fig. 2D) was less than that in the aAD( +) group (63.8 ± 2.8 μm; Fig. 2B). Meanwhile, the cAD( +)/ADFE group demonstrated epidermal hyperplasia and inflammatory cell infiltration (Fig. 2F), and the cAD( +)/EtOH vehicle group showed inflammatory cell infiltration (Fig. 2E). There was no significant difference in epidermal thickness between the cAD( +)/ADFE (44.8 ± 33.1 μm) and cAD( +)/EtOH (42.3 ± 8.5 μm) groups.

Cardiac blood collected at the time of sacrifice showed no statistically significant differences between groups with respect to major hematologic parameters (Table 1). The aAD( +)/ADFE group had higher percentages of neutrophils, monocytes, and basophils, but lower percentages of lymphocytes and eosinophils, than the aAD( +)/EtOH vehicle group, which were not statistically significant. Furthermore, the percentage of eosinophils, monocytes, and basophils in the cAD( +)/ADFE group was lower than that in the cAD( +)/EtOH vehicle group.

**Table 1:** Hematologic parameters in mice with acute or chronic atopic dermatitis treated (or not) with ADFE

Group	WBC (× 10^3^/μl)	Neutrophils (%)	Lymphocytes (%)	Monocytes (%)	Eosinophils (%)	Basophils (%)
aAD(-)	4.75 ± 3.17	23.90 ± 12.59	66.12 ± 14.55	2.93 ± 1.49	5.15 ± 2.13	1.23 ± 1.12
aAD(-)/EtOH	4.03 ± 0.71	22.60 ± 6.51	59.02 ± 14.59	1.18 ± 0.15	2.68 ± 0.99	0.58 ± 0.17
aAD(-)/ADFE	3.54 ± 1.42	25.56 ± 2.38	66.70 ± 1.90	1.43 ± 0.21	4.20 ± 0.46	0.43 ± 0.23
aAD( +)	9.32 ± 6.80	29.05 ± 13.36	62.80 ± 13.85	3.20 ± 0.99	2.10 ± 0.14	1.00 ± 0.28
aAD( +)/EtOH	5.65 ± 0.79	21.27 ± 7.54	69.25 ± 9.96	1.75 ± 0.39	5.55 ± 2.46	0.48 ± 0.10
aAD( +)/ADFE	5.82 ± 1.23	27.60 ± 3.54	63.75 ± 3.89	2.50 ± 0.42	3.95 ± 0.92	0.75 ± 0.35
cAD( +)/EtOH	4.15 ± 2.05	25.80 ± 6.86	64.80 ± 5.69	2.60 ± 0.77	3.60 ± 0.53	0.82 ± 0.50
cAD( +)/ADFE	3.94 ± 1.34	27.96 ± 9.12	64.68 ± 9.32	1.76 ± 0.22	2.56 ± 0.52	0.34 ± 0.05

Blood was collected at the time of sacrifice (Day 28 for the aAD groups and Day 42 for the cAD groups). Data represent the mean ± SD (five mice per group)

ADFE *Artemisia dubia* folium extract, *WBC* White blood cells, *aAD(-), no* Acute atopic dermatitis induction, *aAD(-)/EtOHi *no aAD induction but 10% EtOH vehicle application, *aAD(-)/ADFE *no aAD induction but ADFE treatment, *aAD( +)* aAD induction, *aAD( +)/EtOH* aAD induction with 10% EtOH application, *aAD( +)/ADFE* aAD induction with ADFE treatment, *cAD( +)/EtOH* Chronic AD induction with 10% EtOH application, *cAD( +)/ADFE* cAD induction with ADFE treatment

### Observation of biphasic immune alteration in the present model for induction of aAD or cAD

**Fig. 3:**
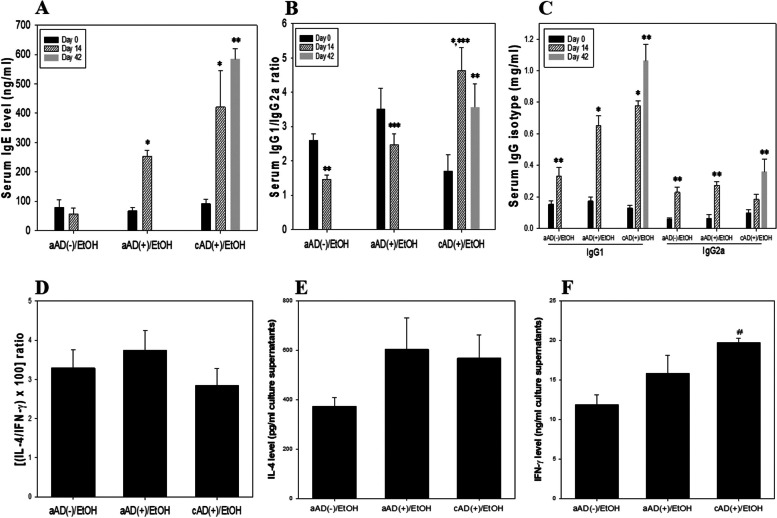
Biphasic immune responses in the aAD and cAD induction models. Serum samples were obtained 1 day prior to (Day 0) the first DNCB sensitization and again at Day 14 following induction. For the cAD groups, an additional sample was collected at Day 42. The IgG1/IgG2a ratio was calculated by dividing serum level of IgG1 by that of IgG2a, and the IL-4/IFN-γ ratio was calculated by dividing the IL-4 level (pg/ml) by the IFN-γ level (pg/ml), followed by multiplication by 100. Single cell suspensions on spleen cells were prepared on Day 28 for the aAD groups and on Day 42 for the cAD groups, and stimulated with immobilized anti-CD3 mAb for 48 h. Data are presented as the mean ± SEM. * indicates significantly different from the aAD(-)/EtOH group at Day 14 and its respective Day 0 level. ** indicates significantly different from its respective Day 0 level. *** indicates significantly different from the aAD(-)/EtOH at Day 14 level. # indicates significantly different from the aAD(-)/EtOH group. The abbreviations were described as in Figs. 1 and 2

IL-4 induces isotype switching to IgG1 and IgE, whereas IFN-γ induces a switch to IgG2a; these antibody profiles reflect the typical predominance of T_H_2 or T_H_1 responses, respectively, in mice [6, 29]. Accordingly, calculating the relative levels of IgG1 versus IgG2a in serum and of IL-4 versus IFN-γ in culture supernatants is used to assess the predominance of T_H_2 or T_H_1 responses, respectively, in individual mice. Serum level of IgE was significantly higher in aAD mice than in non-aAD mice on day 14 and its respective day 0 level (Fig. 3A). This trend was also observed for the cAD group. The IgG1/IgG2a ratio was significantly higher in the aAD group than in the aAD(-)/EtOH group at day 14 (Fig. 3B) and was even higher in the cAD group. Considering the significant upregulation of IgG1 level in the aAD group but no difference of IgG2a level at day 14 compared with those levels in the aAD(-) group at day 14, respectively, IgG1 level seems more influential on the upregulatory IgG1/IgG2a ratio in the aAD group (Fig. 3C). The IL-4/IFN-γ ratio was higher in the aAD group than in the aAD(-)/EtOH group, but lower in the cAD group than in the aAD( +)/EtOH group, at day 42 (Fig. 3D), although the differences were not significant. The continuous increase in IFN-γ production in the cAD group seems to contribute toward the lowered IL-4/IFN-γ ratio at day 42 (Fig. 3F). Similarly, the IgG1/IgG2a ratio was lower in the cAD group on day 42 than in the cAD group on day 14. These findings suggest skewing of the immune response toward T_H_2 at 2 wk post-induction of AD, but this seems to decline by 6 wk despite persistent challenge with DNCB.

### Effect of ADFE on splenic cytokine production

**Fig. 4:**
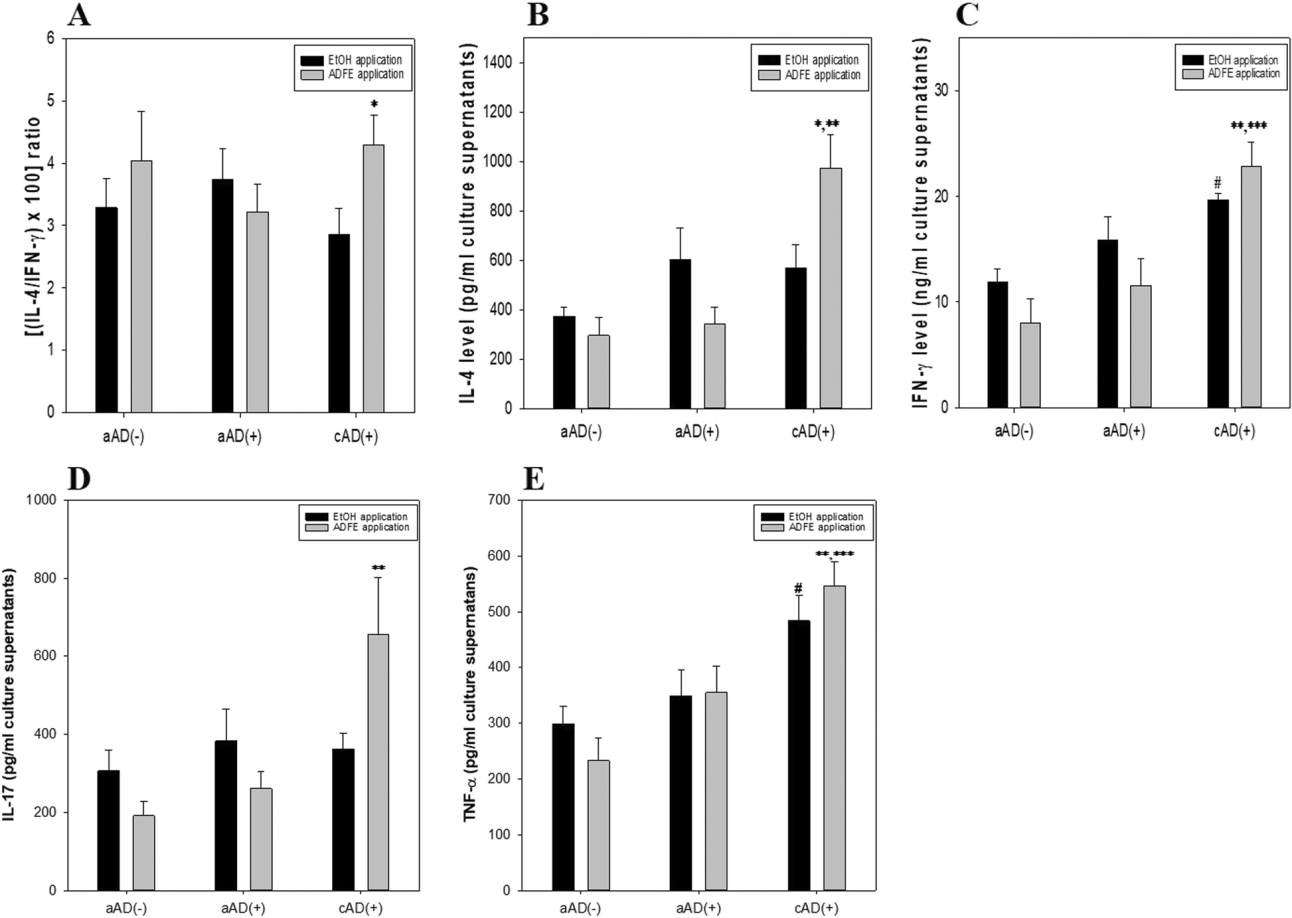
Effect of ADFE on splenic cytokine production. Single splenocyte suspensions were prepared and stimulated as described in Fig. 3. Data are presented as the mean ± SEM. * indicates significantly different from the cAD( +)/EtOH group. ** indicates significantly different from the aAD(-)/ADFE group, and *** indicates significantly different from the aAD( +)/ADFE group. # indicates significantly different from the aAD(-)/EtOH group. The abbreviations were described as in Figs. 1 and 2

ADFE had different effects on the IL-4/IFN-γ ratio in aAD and cAD mice (Fig. 4A). A reduction on the ratio was observed after ADFE treatment of aAD mice, although the difference was not significant. However, a significant increase was observed in ADFE-treated cAD mice compared with the corresponding vehicle control group. Considering the significant upregulation of IL-4 level but no difference of IFN-γ level in the cAD/ADFE group compared with those levels in the corresponding vehicle group, respectively, IL-4 level seems more influential on the upregulatory IL-4/IFN-γ ratio in the cAD group (Fig. 4B & C). These findings suggest that ADFE has a dual function in that it suppresses T_H_2 immune responses in aAD mice and T_H_1-mediated immune responses in cAD mice.

IL-17, an inflammatory cytokine produced by T_H_17 cells, plays a role in pathogenesis of aAD [4]. ADFE reduced IL-17 level in aAD mice; however, the level in cAD mice were higher than that in vehicle control mice (Fig. 4D). Level of another inflammatory cytokine, TNF-α, was higher in aAD mice, and significantly higher in cAD mice, than in non-AD mice (Fig. 4E). However, ADFE had no significant effect on production of TNF-α when compared with the respective vehicle control groups.

### Effect of ADFE on humoral immunity

**Fig. 5:**
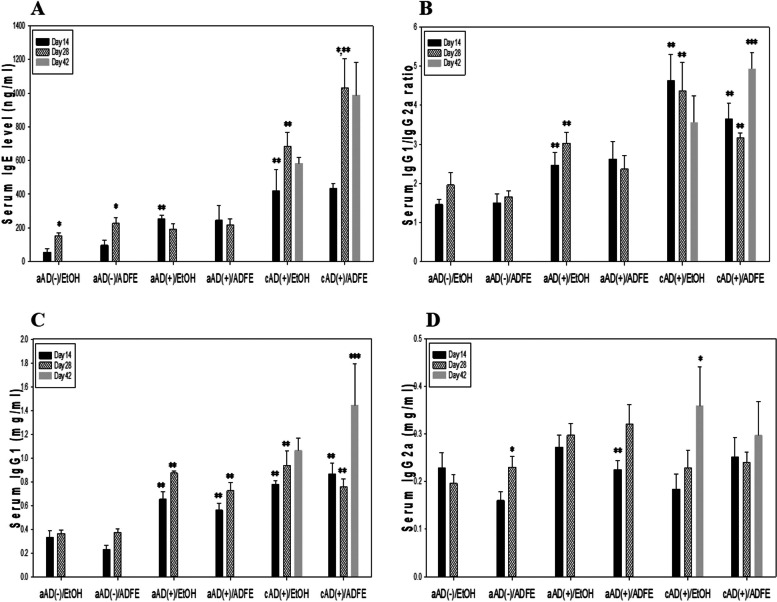
Effect of ADFE on humoral immunity. Serum was obtained at Day 14 and Day 28 following the first DNCB sensitization. An additional collection on Day 42 was made for the cAD groups. The IgG1/IgG2a ratio was calculated as described in Fig. 3. Data are presented as the mean ± SEM. * indicates significantly different from its respective Day 14 level. ** indicates significantly different from the aAD(-)/EtOH or aAD(-)/ADFE group at each day, respectively. *** indicates significantly different from the cAD( +)/ADFE level at Day 28. The abbreviations were described as in Figs. 1 and 2

Serum IgE levels in aAD(-)/EtOH and cAD( +)/EtOH mice were significantly higher on day 28 than in day 14, regardless of ADFE or vehicle treatment; however, this phenomenon was not observed in the aAD( +) group (Fig. 5A). Furthermore, ADFE did not modulate serum IgE levels since similar time-dependent changes in serum IgE levels were observed in both the aAD( +) and cAD( +) groups, regardless of ADFE or vehicle application. The effect of ADFE on the IgG1/IgG2a ratio was similar to that on the IL-4/IFN-γ ratio. In the cAD( +)/ADFE group, a significant increase in the IgG1/IgG2a ratio was noted on day 42 compared with days 14 and 28 (Fig. 5B), whereas the ratio in the aAD( +)/ADFE group was similar between the day 14 and day 28. Serum IgG1 level in the cAD( +)/ADFE group demonstrated the same alteration pattern as the IgG1/IgG2a ratio, whereas such observation was not apparent in the level of serum IgG2a (Fig. 5C & D). These findings could imply in terms of humoral immunity that ADFE has a potential to suppress T_H_1-mediated progression of AD at the later chronic stage through upregulation of T_H_2 reactivity.

## Discussion

To the best of our knowledge, the present study is the first to demonstrate that *Artemisia dubia* improves AD symptoms by regulating the balance between T_H_2- and T_H_1-mediated immune responses during the acute and chronic phases. The paradigm of biphasic progression of AD has been addressed in human subjects [4, 7]. In the present study, the mouse model of AD induced by DNCB also demonstrated predominance of T_H_2-mediated responses during the acute phase of AD, resulting in upregulated serum IgE levels in mice, and an increased IL-4/IFN-γ ratio in murine spleen cell culture supernatants. The T_H_2 response was less prominent during the chronic phase of AD, despite persistent challenge with DNCB, which suggests an increase in T_H_1 activity. Although, ADFE reduced the IL-4/IFN-γ and IgG1/IgG2a ratios during the acute phase, both increased during the chronic phase (compared with the respective vehicle controls). Overall, the findings imply that ADFE concomitantly suppresses T_H_2 responses during aAD and T_H_1 responses during cAD.

The balance between different T cell subsets plays a major role in the pathogenesis of AD [25, 30, 31]. Early-onset AD is thought to be caused by skewing toward a T_H_2 response. Here, we found that ADFE blocked progression of early aAD by suppressing T_H_2-mediated responses, as indicated by a fall in the IL-4/IFN-γ ratio and the IgG1/IgG2a ratio. In addition, ADFE inhibited the augmentation of T_H_1 reactivity in cAD skin, as demonstrated by rebound of both ratios during the chronic phase. IL-17 plays a certain role in inflammatory response in AD [4]. Even though controversial results reported, IL-17 has been reported more often to be highly expressed in aAD stage but downregulated in cAD stage [32, 33]. This upregulatory expression of IL-17 in aAD stage expression is considered to alleviate the augmented T_H_2 reactivity in aAD phase. Application of ADFE at the early acute phase of AD led to a fall in IL-17 production and an increase in production during the persistent chronic phase, suggesting that ADFE could play a role for inflammatory responses during both phases.

IL-4, a representative T_H_2 cytokine, induces immunoglobulin isotype switching to IgG1, while the T_H_1 cytokine IFN-γ induces isotype switching to IgG2a [13, 28]. An increase in the IgG1/IgG2a ratio reflects the dominance of a T_H_2-mediated immune response observed in early acute AD, which was not observed following application of ADFE for 2 weeks. In addition, a reduced IgG1/IgG2a ratio reflects the dominance of T_H_1-mediated immune responses, typically observed in the persistent chronic group. ADFE application during the chronic AD phase led to a significant increase in the ratio by day 42, strongly suggesting that ADFE suppresses T_H_1 responses. Binding of IgE to antigen-specific FcεRI receptors on mast cells triggers degranulation of mast cells, whereas concurrent release of histamine causes itching, increased vascular permeability, and hypersensitivity [11, 34, 35]. We did not find that ADFE had a significant effect on serum IgE levels, suggesting that ADFE might not prevent IgE binding to mast cells. However, it may be postulated that ADFE may inhibit binding of histamine to its receptor, thereby controlling the histamine-mediated clinicopathologic effects. Histamine is a well-known mediator of cutaneous inflammatory response implicated with exacerbations of eczematous lesions [36, 37]. Therefore, the present pathologic results demonstrating amelioration of epidermal thickness and improvement of cutaneous condition in the aAD( +)/ADFE group could suggest the potential function of ADFE on the aAD. This possibility should be investigated to further elucidate the mechanism by which ADFE ameliorates AD.

T_H_2 cytokines play a key role in the differentiation, recruitment, and effector function of eosinophils, which are an important mediator of AD [38]. The present study demonstrated a tendency toward decreasing eosinophil numbers in the blood of mice with aAD and cAD after treatment with ADFE. Considering this result together with the immunoregulatory potency of ADFE on AD-related cytokines and immunoglobulin isotypes described above, ADFE appears to regulate the immune system on multiple levels to ameliorate AD. Macroscopic observation of skin sections from ADFE-treated mice with early aAD indicated a reduction in clinical symptoms of eczema and erythema, and concomitant slowing of progression of AD-like skin lesions, which was not observed in the persistent cAD group. These findings suggest that ADFE can provide symptomatic relief during the early phase of AD, although the finding that the skin is entirely xerotic and lichenified during cAD suggests that recovery might be limited [39]. Previous studies show that epidermal hyperplasia and increased inflammatory cell infiltration are major histological findings in those with AD [13, 28]. The marked decrease in epidermal thickness in AD-like skin lesions following treatment with ADFE for 2 weeks suggests that the compound mitigates hyperplasia during the acute phase.

Previous studies on *Artemisia dubia* focused on its anti-microorganismal or anti-cancerous properties in vitro [18, 20]. Phytochemical investigation of *Artemisia dubia* extract revealed the presence of bioactive constituents such as flavonoids, triterpenoids, phenolics, and steroids [19, 20]. These bioactive components might underpin the anti-inflammatory action of *Artemisia dubia*; indeed, flavonoids, phenolics, and steroids are anti-inflammatory [21, 40]. ADFE could contribute toward alleviation of atopic dermatitis progression through controlling inflammatory responses in AD. ADFE application may downregulate the immune pathogenesis implicated with T_H_2 predominance in acute AD phase, which could be supported by the present results with lowered ratios of IL-4/IFN-γ and IgG1/IgG2a in the acute phase. In addition, ADFE application could concomitantly reverse the immune-alteration related with T_H_1 predominance in chronic AD phase, in that those ratios were upregulated in cAD/ADFE group.

## Conclusions

The present study revealed the efficacy of ADFE on immunomodulation implicated with induction and/or progression of atopic dermatitis-like skin lesions in mice. ADFE application on dorsal skin lesions mitigated the epidermal hyperplasia in the acute phase of AD, and eosinophilia was relieved in both acute and chronic phase. Further investigation seems necessary to delineate mechanisms involved with ADFE’s dual functions in controlling T_H_2 or T_H_1 reactivity in biphasic progression of AD. In addition, it is preferred to identify bioactive components in ADFE responsible for its immunomodulatory functions on alleviation of AD.

### Supplementary Information

**Additional file 1.** Macroscopic images of ADFE-treated mouse skin after induction of atopic dermatitis by application of DNCB. (A) Skin lesion in aAD mice following sensitization for 1 week, and challenge for 1 week with 1% or 0.2% DNCB. (B) Improved skin appearance in mice with aAD treated for 2 weeks ADFE. (C) Lichenified skin lesions in mice with cAD treated with 10% ethanol. (D) Lichenified skin in cAD mice treated with AVFE for 4 weeks (cAD was induced by DNCB challenge for 5 weeks). ADFE, Artemisia dubia folium extract; DNCB, 2,4-dinitrochlorobenzene; aAD, acute atopic dermatitis; cAD, chronic AD.

## Data Availability

The data that support the findings of this study are available from the corresponding author, Dr. Yong Heo, upon reasonable request under permission of the DragonImmuno Inc. and the Industry Academic Cooperation Foundation, Daegu Catholic University.

## Abbreviations

aAD: Early phase acute atopic dermatitis
AD: Atopic dermatitis
ADFE: *Artemisia dubia* folium extract
ANOVA: One-way analysis of variance
cAD: Persistent chronic atopic dermatitis
DNCB: 2, 4-Dinitrochlorobenzene
ELISA: Enzyme Linked Sorbent Assay
IFN-γ: Interferon-gamma
IL-: Interleukin-
T_H_1: Type-1 helper T cell
T_H_2: Type-2 helper T cell
TNF-α: Tumor necrosis factor-alpha
