# Supplementary figures and images for: Evaluation of Artemisia dubia folium extract-mediated immune efficacy through developing a murine model for acute and chronic stages of atopic dermatitis
Source: Lab Anim Res. 2024 Apr 7;40:13. doi: 10.1186/s42826-024-00201-x (PMC10999079; doi:10.1186/s42826-024-00201-x)

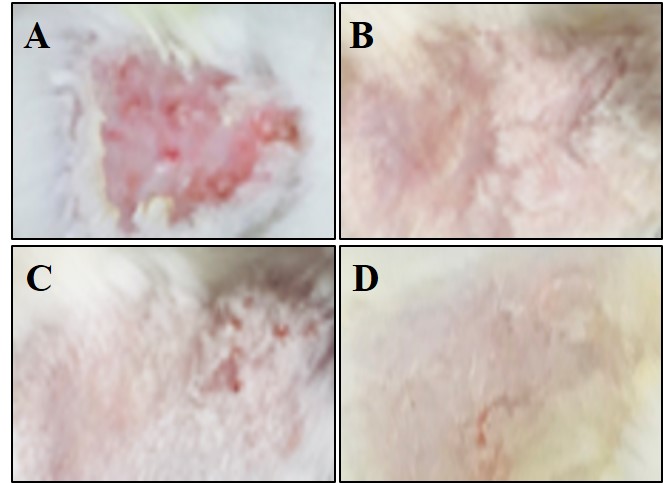

Supplement: Supplementary file 1 — Additional file 1. Macroscopic images of ADFE-treated mouse skin after induction of atopic dermatitis by application of DNCB. (A) Skin lesion in aAD mice following sensitization for 1 week, and challenge for 1 week with 1% or 0.2% DNCB. (B) Improved skin appearance in mice with aAD treated for 2 weeks ADFE. (C) Lichenified skin lesions in mice with cAD treated with 10% ethanol. (D) Lichenified skin in cAD mice treated with AVFE for 4 weeks (cAD was induced by DNCB challenge for 5 weeks). ADFE, Artemisia dubia folium extract; DNCB, 2,4-dinitrochlorobenzene; aAD, acute atopic dermatitis; cAD, chronic AD. [file 42826_2024_201_MOESM1_ESM.jpg]
